# A single active site in the *mariner* transposase cleaves DNA strands of opposite polarity

**DOI:** 10.1093/nar/gkx826

**Published:** 2017-09-19

**Authors:** Corentin Claeys Bouuaert, Ronald Chalmers

**Affiliations:** School of Biomedical Sciences, University of Nottingham, Queen’s Medical Centre, Nottingham NG7 2UH, UK

## Abstract

The RNase H structural fold defines a large family of nucleic acid metabolizing enzymes that catalyze phosphoryl transfer reactions using two divalent metal ions in the active site. Almost all of these reactions involve only one strand of the nucleic acid substrates. In contrast, cut-and-paste transposases cleave two DNA strands of opposite polarity, which is usually achieved via an elegant hairpin mechanism. In the *mariner* transposons, the hairpin intermediate is absent and key aspects of the mechanism by which the transposon ends are cleaved remained unknown. Here, we characterize complexes involved prior to catalysis, which define an asymmetric pathway for transpososome assembly. Using mixtures of wild-type and catalytically inactive transposases, we show that all the catalytic steps of transposition occur within the context of a dimeric transpososome. Crucially, we find that each active site of a transposase dimer is responsible for two hydrolysis and one transesterification reaction at the same transposon end. These results provide the first strong evidence that a DDE/D active site can hydrolyze DNA strands of opposite polarity, a mechanism that has rarely been observed with any type of nuclease.

## INTRODUCTION

RNase H defines a large family of enzymes that share a core structural fold. These enzymes are involved in variety of processes including transposition, DNA replication, repair and recombination, RNA interference and CRISPR-Cas9 transposition (1–5). These enzymes have three or four acidic amino acids that coordinate two divalent metal ions in the active site. The catalytic activity is usually confined to a single strand of their respective nucleic acid substrates.

The simplest transposition reaction is exemplified by phage Mu. The transposase hydrolyzes one strand at the transposon end and integrates the resulting 3′-OH at the target site (6). A single active site is therefore sufficient. In contrast, during cut-and-paste transposition, both DNA strands at the transposon end must be cleaved. Some cut-and-paste transposases conform to the single-strand activity rule and have recruited a second protein to perform the second cleavage event. In Tn*7*, the second strand is cleaved by a separate subunit related to restriction endonucleases (7). Nevertheless, many transposases are able to perform double strand cleavage using a single active site (2,8). This is achieved via an elegant mechanism involving a DNA hairpin intermediate. In Tn*10*, the first nick exposes the 3′-OH at the end of the transposon. In a reaction that foreshadows the final integration step, this group acts as a nucleophile to cleave the opposite strand, generating the hairpin on the transposon end and separating the transposon from the donor site (9–11). The hairpin is resolved by a second hydrolysis reaction, yielding the 3′-OH and 5′-phosphate groups on the cleaved transposon end (12,13). In the RAG1/2 recombinase and some transposons, the hairpin is on the flanking donor side of the break but the steps are otherwise similar (14,15).

In the *IS630-Tc1-mariner* (ITm) family of transposons the first nick exposes the 5′-phosphate, which is usually recessed two or three bases within the element. This is followed by a second nick that generates the 3′-OH at the transposon end (16–19). However, this second strand cleavage reaction does not appear to involve a hairpin intermediate (16,20,21).

Until the discovery of the peculiar case of *mariner*, the hairpin strategy was assumed to be the universal mechanism by which homomeric transposases generate double-strand breaks at their asymmetric transposon ends. Although the hairpin is absent in *mariner*, the cleavage mechanism remains poorly understood. It has been widely assumed that the chirality of the phosphodiester backbone, and the identities of the bases surrounding the cleavage site, dictate that the active site of a given nuclease will only accommodate a strand with a particular polarity. This has been an important consideration in understanding the mechanism of monomeric restriction endonucleases. The best-known example is probably FokI, which was found to obey the single polarity rule: a DNA-bound monomer recruits a second subunit by weak protein–protein interactions (22,23). This precedent, and others, suggested that double strand cleavage in *mariner* might happen through the sequential action of separate active sites, either through a tetrameric transposase or through exchange of subunits within a dimer during catalysis. Indeed, phage Mu and the foamy virus integration provided an alluring precedent for a tetrameric transpososome, although in both cases only two of the four DNA strands are cleaved and only two of the four subunits are involved in catalysis (24,25).

Previous studies have attempted to shed light onto this question by characterizing protein–DNA complexes formed between *mariner* transposases and the transposon ends (18,26–28). Generally, these have been difficult to interpret and have often led to contradictory conclusions. Electrophoretic mobility shift assay (EMSA) experiments with *Mos1* provided evidence for two structural isoforms of a transposase-dimer complexed with a single transposon-end, which appeared to mature into a tetrameric paired-ends complex (PEC) (26). Earlier experiments with *Himar1* also identified isoforms of the single-end complex, which appeared to arise from the loss of weak protein–protein interactions during electrophoresis (18).

One difficulty in studying the mechanism of *mariner* is that the synaptic complex, wherein catalysis takes place, is notably absent in EMSA analyses for *Mos1, Himar1* and *Hsmar1*. It is clear, though, that this complex must exist because *mariner* cleaves single-end substrates inefficiently compared to double-ended substrates. This indicates that the PEC is a prerequisite for cleavage (29). In addition, transposon integration is concerted and intramolecular integration products retain topological information from the substrate. This shows that the synaptic complex must be maintained throughout catalysis (29). Nevertheless, the absence of the PEC in EMSAs has hindered the characterization of transpososome dynamics throughout the reaction.

More recently, a series of crystal structures of *Mos1* complexes assembled with pre-cleaved or partially cleaved substrates have revealed a dimeric PEC (30–32). However, structural studies do not capture dynamics and cannot rule out models in which subunits change their roles or positions during the reaction (33). The stoichiometry of the pre-cleavage complexes, the dynamics of the PEC throughout catalysis and the role of individual subunits in the reaction thus remain unclear.

Here, we have tested various cleavage models using the human *mariner*-family transposon *Hsmar1* (34,35). We show that all the chemical steps of *mariner* transposition are carried out by one transposase dimer. One monomer performs two sequential strand cleavages and one strand transfer reaction at the same transposon end. These findings exclude models for sequential hydrolysis that involve loosely bound subunits or subunit exchange between transposon ends and suggest that the two DNA strands might engage the active site with the opposite polarity.

## MATERIALS AND METHODS

### Plasmid substrates and expression vectors

All the expression vectors used the reconstructed transposase sequence codon-optimized for expression in *Escherichia coli* (35). The expression vector for the *^MBP^Hsmar1* transposase is pRC880, which is derived from pMAL-c2x (35). The expression vector for *^2MBP^Hsmar1* (pRC1116) was generated by cloning a polymerase chain reaction-amplified fragment from pRC880 coding for *^MBP^Hsmar1* between the XmnI and BamHI sites of pMAL-c2x. The expression vector for *^TrxA^Hsmar1* (pRC1108) was generated by cloning the *Hsmar1* transposase gene between the BamHI and HindIII sites of pET-32a(+). The expression vector for the long-short (LS) heterodimer (pRC1122) was constructed by cloning the gene coding for the *Hsmar1* transposase between the EcoRI and XbaI sites of pMAL-c2x to obtain pRC1123, followed by the insertion of a XbaI and HindIII fragment from pRC1108, which contains the gene coding for *^TrxA^Hsmar1* together with its ribosome binding site (RBS). The expression vector for the single-chain dimer (pRC1127) was modified from the LS heterodimer expression vector by replacing the intervening sequence between *^MBP^Hsmar1* and *^TrxA^Hsmar1*, including the stop codon of *^MBP^Hsmar1* and the RBS, with a sequence coding for an 18 amino acid flexible linker (SRGGGSEGGGSEGGSGTS). The resulting sequence provides a 187 amino-acid linker between the C-terminus of the first subunit and N-terminus of the second subunit, which includes, in addition to the 18 amino-acid sequence above, a TrxA tag, six histidines, a thrombin recognition site, a S-tag and an enterokinase recognition site. This provides ample sequence length to span the ∼60 Å between the opposite ends of the two subunits predicted from the *Mos1* PEC structure (30–32), without introducing significant strain. Other expression vectors include pRC1113 for the *^MBP^Hsmar1-D155A* mutant; pRC1144 and pRC1145 for the single-chain dimers with a D155A mutation in the first and second subunits, respectively; pRC1146 and pRC1147 for the single-chain dimers with a R104A mutation in first and second subunits, respectively. Point mutants were generated by QuickChange mutagenesis. Transposition reactions contained the inverted-repeat substrate pRC650 that provides a 1.7 kb transposon and a 3 kb plasmid backbone (35). Gel shift analyses used a linear fragment that carried an *Hsmar1* transposon end generated from plasmid pRC919 (29). The sequences of the fragments are as follows (with transposon end sequence underlined and the flanking TA dinucleotide italicized): short (96 bp): CCGGGCTGCAGGAATTC*TA*TTAGGTTGGTGCAAAAGTAATTGCGGTTTT GGATCCCAAGCTTCTTCTAGAGGTACCGCATGCGATATCGAGCTCTC; long (162 bp): GCGGTGGCGGCCGCTCTAGAACTAGTGGATCCCCCGGGCTGCAGGAATTC*TA*TTAGGTTGGTGCAAAAGTAATTGCGGTTTT GGATCCCAAGCTTCTTCTAGAGGTACCGCATGCGATATCGAGCTCTCCCGGGAATTCGATATCAAGCTTATCGATACCGT.

### Transposase purifications

All maltose-binding protein (MBP)-fusion transposases were purified as described previously (35). For *^TrxA^Hsmar1*, the protocol was adapted for purification on Ni-NTA Superflow resin. Briefly, *E. coli* cells overproducing transposase were harvested by centrifugation and resuspended in Ni-buffer (50 mM HEPES pH 7.5, 500 mM NaCl, 10 mM imidazole, 0.1% Triton X-100 reduced, 10% glycerol). After cell lysis and centrifugation, the supernatant was loaded onto a disposable column containing Ni-NTA Superflow resin (Qiagen). The column was washed with Ni-buffer and eluted with Ni-buffer containing 150 mM imidazole. The LS heterodimer was first purified on amylose resin then the protein was diluted in Ni-buffer and purified on Ni-NTA Superflow resin. Except for the LS heterodimer all proteins were further purified by ion-exchange chromatography on a MonoS HR5/5 column (Amersham Pharmacia). Purified proteins were flash frozen and stored at −80°C.

### Size exclusion chromatography

Gel filtration experiments were performed using a Superdex 200 10/300 GL column (GE Healthcare) in GF buffer (25 mM HEPES pH 7.5, 200 mM NaCl, 2 mM dithiothreitol (DTT), 5 mM ethylenediaminetetraacetic acid (EDTA), 0.1% Triton X-100 reduced). Typically, 100 μl samples at concentrations of 0.5–2 mg/ml were injected onto the column with a flow rate of 0.4 ml/min. Proteolysis experiments used 1 μg of protease to cleave 50 μg of fusion protein and were performed for 1 h to overnight at 4°C or at room temperature.

### *In vitro* transposition assay

Transposition assays were performed essentially as described before (35). Transposase concentrations were carefully adjusted to optimal levels because an excess of transposase inhibits the reaction while lower concentrations do not allow complete consumption of the substrate (28). Unless stated otherwise, a 50 μl reaction contained 6.7 nM (1 μg) of the plasmid substrate pRC650 and 20 nM of transposase in 20 mM Tris–HCl buffer pH 8, 100 mM NaCl, 10% glycerol, 2 mM DTT and 2.5 mM MgCl_2_. After 4 h at 37°C, reactions were stopped with 25 mM EDTA and 1% sodium dodecyl sulphate and heated at 75°C for 30 min. DNA was recovered by ethanol precipitation, resuspended in TE buffer and 400 ng was loaded in each lane of a TBE-buffered 1.1% agarose gel. After electrophoresis, gels were stained with ethidium bromide and photographed. For analyses of transposition products treated with a restriction endonuclease, the products of transposition reactions were digested with BsaHI and 3′-labeled with α-^32^P-dCTP and the Klenow enzyme. Products were separated on a TBE-buffered 1.1% agarose gel (native) or an alkaline (50 mM NaOH, 1 mM EDTA) 1.5% agarose gel (denaturing), the gels were dried and recorded on a Fuji phosphorimager.

### EMSA

DNA fragments encoding *Hsmar1* transposon ends were prepared by digesting pRC919 with XmaI (short, 96 bp) or SacII and AccI (long, 162 bp) and labeled with α-^32^P-dCTP and the Klenow enzyme. Unless stated otherwise each 20 μl reaction contained 250 ng of non-specific plasmid DNA, 2 nM labeled substrate and 10 nM transposase. Complexes were assembled for 1 h in buffer containing 20 mM HEPES pH 7.5, 100 mM NaCl, 2 mM DTT, 10% glycerol, 5% DMSO, 5 mM CaCl_2_ and 250 μg/ml BSA. Products were separated on a 5% Tris-acetate-EDTA polyacrylamide gel. The gels were dried and imaged by autoradiography.

## RESULTS

### The *Hsmar1* transposase is a dimer with a slow rate of subunit exchange

We set out to determine the number and the role of subunits required for transposon cleavage. To address this, we first determined the oligomeric state of the *Hsmar1* transposase in solution. Previous studies have reported that *mariner* transposases behave either as monomers (18), mixtures of monomers and dimers (26,36) or dimers (20,35). We set out to address this unequivocally for the *Hsmar1* transposase.

**Figure 1.**
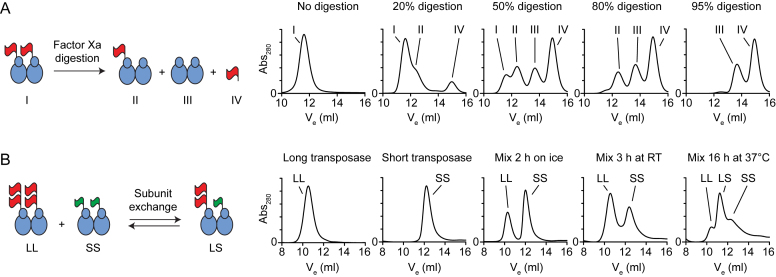
*Hsmar1* transposase is a dimer with a slow rate of subunit exchange. (**A**) Size exclusion chromatography analysis of Factor Xa proteolysis reactions of purified ^MBP^*Hsmar1* transposase. The linker region between the MBP tag (red flag) and *Hsmar1* carries a cleavage site for Factor Xa protease. The four products expected from partial digestions of a dimeric transposase are illustrated in the cartoon (I–IV). The MBP tag approximately doubles the size of transposase (the ^MBP^*Hsmar1* monomer is 83.5 kDa). Factor Xa protease, which was present at a concentration of 1 μg per 50 μg of transposase, has an apparent mass equivalent to MBP (∼43 kDa) and therefore slightly contributes to peak IV. (**B**) Size exclusion chromatography analysis of mixtures of long (LL) and short (SS) transposase dimers. A long monomer (*^2MBP^Hsmar1*) was 126 kDa and a short monomer (*^TrxA^Hsmar1*) was 58 kDa.

We purified the *Hsmar1* transposase as a fusion with an N-terminal MBP, which approximately doubles its size. The linker between the MBP tag and transposase has a cleavage site for Factor Xa protease. A partial digestion of a dimeric fusion protein would generate four species of distinct molecular sizes, which are the transposase dimers with two, one or no MBP tags, and the monomeric MBP liberated upon proteolysis (Figure 1A, cartoon). We performed a set of gel filtration analyses with samples subjected to increasing levels of proteolysis. A single peak was detected in the absence of Factor Xa (No digestion, peak I). Gradual proteolysis was accompanied by the accumulation of the MBP tag (peak IV) and the fully cleaved transposase (peak III). The intermediate product that carries a single tag on a dimeric transposase (peak II) was detected at intermediate levels of proteolysis but disappeared when proteolysis was complete. This demonstrates that the *Hsmar1* transposase is a dimer in solution.

To evaluate the rate of subunit equilibration between transposase dimers we performed a set of gel filtration analyses with mixtures of long (LL) and short (SS) dimers (Figure 1B). The long and short transposases had a double-MBP and a TrxA tag, respectively. The large size difference between the tags increased the resolution of the gel filtration and facilitated the detection of the various species. Long and short transposase dimers were analyzed separately or as equimolar mixtures after being left on ice for 2 h, at room temperature for 3 h, or at 37°C for 16 h. No intermediate peak was observed when proteins had been mixed on ice for 2 h. After 3 h at room temperature, two peaks started to overlap suggesting that some subunit exchange had occurred. After overnight incubation at 37°C, the subunits had reached equilibrium and the species were present at the expected 1:2:1 ratio (Figure 1B, rightmost panel). The transposase dimers are therefore relatively stable but will exchange subunits after extended incubation.

### The stoichiometry of the single-ended complexes

**Figure 2.**
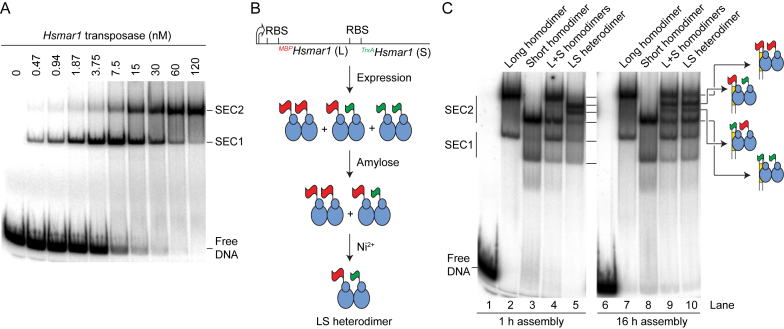
Stoichiometry of the single-ended complexes. (**A**) EMSA analysis of *Hsmar1* reveals two single-ended complexes. The *^MBP^Hsmar1* transposase was titrated in the presence of 2 nM of a 96 bp linear substrate that carried a transposon end. An autoradiograph of a native polyacrylamide gel is shown. (**B**) Illustration of the purification strategy of the LS heterodimer. One long (L) and one short (S) copy of the *Hsmar1* transposase gene were cloned in direct repeat downstream of an inducible promoter (thick arrow). The long monomer is expressed as a fusion with MBP (red flag), which can be purified on an amylose resin. The short monomer is expressed as a fusion with TrxA (green flag), which also carries a His6-tag that can be purified on a nickel-ion chelating resin. Transposase overexpression produces homodimers and the heterodimer. The heterodimer can be selectively purified by sequential affinity chromatography. Long and short subunits are 83 kDa (*^MBP^Hsmar1*) and 58 kDa (*^TrxA^Hsmar1*) respectively. (**C**) The stoichiometry of the single-ended complexes. Complexes were assembled between a linear DNA fragment carrying a transposon end and different versions of the transposase. Each 20-μl reactions contained 40 fmol substrate (2 nM) and 10 nM transposase. Complexes were assembled for the indicated amount of time at 37°C and separated on a 5% native polyacrylamide gel.

We went on to characterize the complexes formed between the *Hsmar1* transposase and the transposon end. A linear DNA fragment encoding the transposon end was titrated with transposase and the complexes were analyzed by gel electrophoresis (Figure 2A). This revealed two single-ended complexes, labeled SEC1 and SEC2, which are similar to the complexes usually observed with other *mariner* transposases (18,26,28,37).

To assess the transposase stoichiometry, we began by generating LL, SS and LS versions of the transposase dimer (Figure 2B). These differed in size according to whether they have a MBP or a His-tag fusion on the N-terminus. To generate the heterodimer, the MBP- and His-tagged derivatives were co-expressed and purified by sequential affinity-chromatography steps. Protein–DNA complexes were assembled at 37°C for 1 h and analyzed in the EMSA (Figure 2C, left panel). The long and the short homodimers produced SEC1 and SEC2 complexes with the expected mobility, and when they were mixed no new bands appeared (lane 4). On the other hand, the LS heterodimer produced a novel doublet at a position intermediate to the long-SEC2 and the short-SEC2 (lane 5). This demonstrates that SEC2 contains a dimer of transposase. We interpret the doublet as structural isoforms of SEC2, in which either the long or the short subunit is bound in *cis* to the transposon end, and are separated on the gel because of their asymmetry. In addition, the LS heterodimer produced bands corresponding to long-SEC1 and short-SEC1 (lane 5). This demonstrates that SEC1 contains a monomer of transposase. We have previously shown that SEC1 arises from dissociation of the PEC during electrophoresis (18,26,28,37). SEC1 is therefore probably not an intermediate of the transposition reaction.

The EMSA was repeated with the incubation extended from 1 to 16 h (Figure 2C, right panel). The LL and the SS homodimers gave the same pattern of bands (lanes 7 and 8). However, the mixture of LL and SS homodimers (lane 9), and the purified LS heterodimer (lane 10), generated the superposition of all the respective bands in lanes 4 and 5. This agrees with the results above, which show that transposase dimers reassemble if provided long incubation times.

### A single-chain dimer allows visualization of the paired-ends complex

Since the PEC is undetectable in the EMSA, we created a single-chain dimer in which the N- and C-termini of the subunits are connected by an amino acid linker as a way to stabilize the subunit interface during electrophoresis. The protein was active in an *in vivo* assay and gel filtration analysis showed that the protein did not form dimers-of-dimers at a detectable level (Supplementary Figure S1).

**Figure 3.**
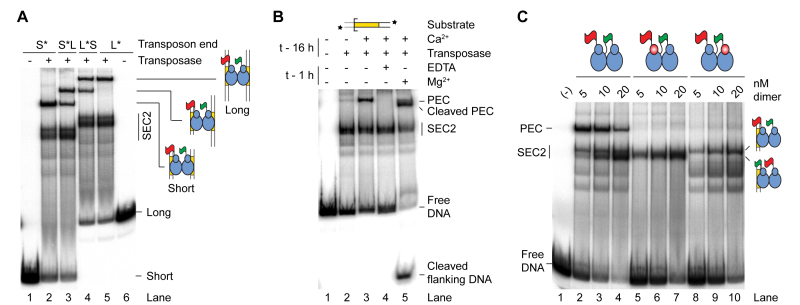
Single-chain dimer and the paired ends complex. (**A**) Identification of the PEC using the transposon end ‘long-by-short’ strategy. Complexes were formed with the single-chain dimer in the presence of combinations of long (L) and short (S) transposon ends that were either unlabeled or radioactively labeled, as indicated by the asterisk. The presence of two transposon ends in the PEC is revealed by the appearance of a band of intermediate mobility in reactions that contain a mixture of long and short substrates. (**B**) The single-chain dimer assembles a Ca^2+^-dependent PEC that is catalytically active. Complexes were assembled overnight in the presence or absence of Ca^2+^. Where indicated, EDTA or the catalytic metal ion Mg^2+^ was added for 1 h before the complexes were separated by electrophoresis. (**C**) The PEC most likely contains a single transposase dimer. Complexes were assembled with the wild-type single-chain dimer or mutant single-chain dimers that carried a mutation in the DNA-binding domain (R104A) of one of the transposase subunits (red ovals). The two structural isoforms of SEC2, in which the first (MBP-tagged) or the second (TrxA-tagged) subunit of the dimer is bound in *cis* to the transposon end, are indicated. The decreasing amount of PEC at the expense of SEC2 with increasing transposase concentration is a predicted feature of our model of *mariner* autoregulation (28) (see Figure 6A). For a characterization of the single-chain dimer see also Supplementary Figure S1.

Analysis of the single-chain dimer by EMSA revealed a new band migrating above SEC2 (Figure 3A, lane 2). To determine if this corresponded to the PEC, we used a combination of long and short transposon ends. When the radioactively labeled short transposon-end was supplemented with an unlabeled long transposon-end, a new band appeared above the putative short-PEC (lane 3). SEC2 was unchanged, as expected for a complex containing one transposon end. In the reciprocal experiment, in which a labeled long transposon-end was supplemented with an unlabeled short transposon-end, a new band appeared below the putative long-PEC (lane 4). Since the new bands in lanes 3 and 4 migrate at the same position, it suggests that they correspond to a PEC with one short and one long transposon end.

Since a covalent link has been introduced between transposase monomers, one possibility might be that the putative PEC-band represents two inactive SEC1 complexes held together by the linker. To address whether the complex behaves like a *bona fide* PEC, we asked whether the complex is dependent on the presence of Ca^2+^. Indeed, we previously showed that Ca^2+^ supports the assembly of the transpososome, but not catalysis (28,35). When Ca^2+^ was omitted from the assembly reaction or when pre-assembled complexes were challenged with EDTA prior to electrophoresis, the PEC was no longer detected (Figure 3B, lanes 2 and 4). To test the activity of the single-chain dimer we assembled the complex in the presence of Ca^2+^ then added the catalytic metal ion (Mg^2+^) 1 h before the start of the EMSA. In the presence of Mg^2+^, the complex shifted down the gel and a fast-migrating cleavage product was detected (Figure 3B, lane 5). This shows that prior to electrophoresis the complex is competent for cleavage and represents a *bona fide* PEC. The minor shift in electrophoretic mobility between the pre- and post-cleavage PECs suggests that their protein content remains unchanged. Since the dimeric stroichiometry of the post-cleavage PEC already received strong support (30–32), this experiment is consistent with a dimeric pre-cleavage PEC. Indeed, the dimeric stoichiometry of the PEC is also consistent with the monomeric stoichiometry of SEC1 into which it dissociates (above and (28)).

The *Hsmar1-R104A* mutation weakens the interactions between the transposase and DNA (28). The residue lies within the second helix-turn-helix motif of the transposase, which is important for the recognition of the transposon end (30,38,39). This mutation was introduced separately in the two subunits of the single-chain dimer. Complexes were assembled with these mutants and analyzed by EMSA (Figure 3C). The PEC observed with the wild-type (WT) single-chain dimer was absent with the two mutants. This further suggests that the PEC does not contain additional structural units. Indeed, in a tetrameric PEC, only two of the four R104 residues would be in sequence-specific interactions with the ends and mutating one subunit per dimer would probably not abolish the assembly of the complex. Therefore, the most likely interpretation is that the two transposase monomers are involved in sequence-specific interactions with the transposon ends and are necessary and sufficient for the assembly of an active PEC.

In addition, SEC2, which migrates as a doublet with WT single-chain dimer, appears as a single band with the two mutants. The top and the bottom bands of the doublet disappeared when the mutation was introduced in the first (long) and second (short) subunit of the single-chain dimer, respectively. This identifies the two structural isoforms of SEC2, as illustrated (Figures 2C and 3C).

The results presented so far identify the pre-cleavage complexes and suggest a model whereby a transposase dimer binds one transposon end to form SEC2, which then matures into a dimeric PEC (18,26,28,37). That is, the transpososome assembles asymmetrically by sequential binding of a transposase dimer to two transposon ends. These results do not, however, reveal the role of individual monomers in the cleavage reaction and cannot exclude the possibility that additional subunits are involved.

### Models of the transposition reaction

**Figure 4.**
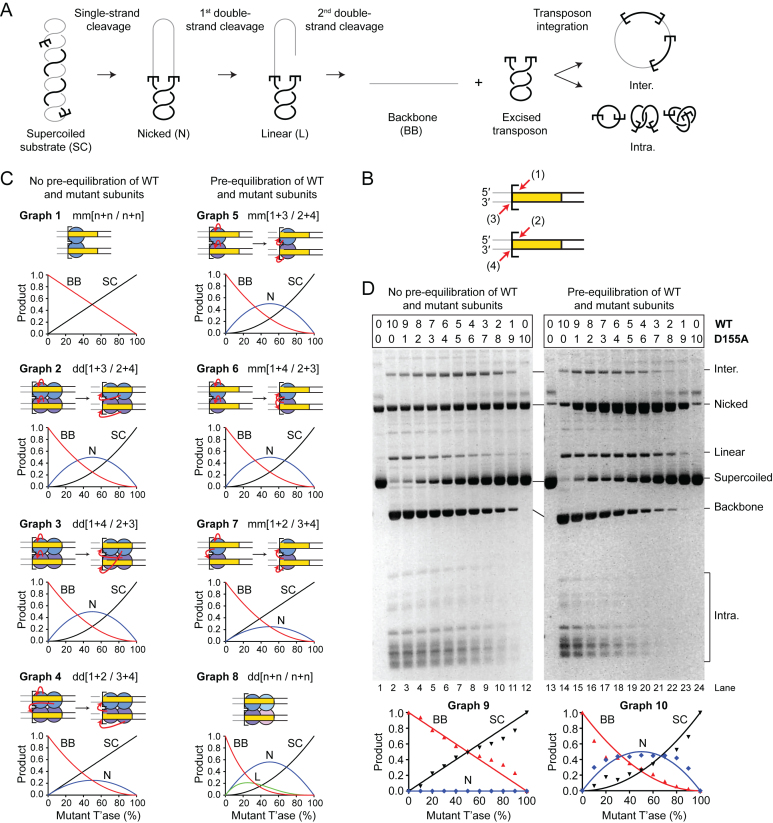
A single transposase dimer performs all the catalytic steps of transposition. (**A**) An illustration of the plasmid transposition assay. (**B**) The four DNA strand cleavages. Nicks 1 and 2, and nicks 3 and 4 are indistinguishable from each other because of the rotational symmetry of the complex. Note that the order of strand cleavages appears to be a kinetic constraint rather than an absolute mechanistic constraint. See also Supplementary Figure S2. (**C**) Simulations of transposase mixing reactions. The different models for the roles of individual subunits during cleavage are illustrated, together with graphs predicting the outcomes of transposition reactions with various amounts of WT and catalytically inactive transposases. When the cleaved strand is given as ‘n’ it indicates that all models predict the same outcome irrespective of which subunit cleaves which strand. Two scenarios are considered: (left panel) reaction mixtures contain only WT and mutant homodimers; (right panel) reaction mixtures contain statistical distributions of WT and mutant homodimers and heterodimers. The predicted outcomes were calculated for models involving two transposase active sites (one dimer) or four transposase active sites (two dimers). A similar strategy was previously used to determine the role of individual subunits in the phage Mu and Tn*10* transpososomes (8,55). See also Supplementary Figures S3 and 4. (**D**) Transposition reactions with mixtures of WT and catalytically inactive (D155A) transposases. In the left panel, transposase dimers were not allowed to redistribute prior to the reaction, which therefore contain essentially homodimers only. In the right panel, transposase dimers were allowed to redistribute prior to the reaction, which therefore contained homodimers and heterodimers. Photographs of ethidium bromide agarose gels are shown. The bands of the intermediates and products of transposon excision (SC, N, L and BB) were quantified and plotted (bottom panels). Plots display the experimental results (dots) and the predicted outcome of the two active sites models m/m[1+3/2+4] or m/m[1+4/2+3] (lines), which best fits the data. Note that the contaminating nicked substrate does not contribute significantly to the reaction because it reacts much more slowly than the supercoiled substrate (29). Numbers given above the gel lanes are transposase ratios. The lack of catalytic activity of the *Hsmar1-D155A* transposase is evident from a comparison of the plasmid preps (lanes 1 and 13) with reactions that contained only the mutant transposase (lanes 12 and 24).

To address the number of subunits required for transposition, and their respective roles in the reaction, we devised a strategy based on how the ratio of active and catalytically inactive protein affect the distribution of products. We used a supercoiled substrate for the reaction because almost all of the intermediates and products can be monitored easily (Figure 4A). A single strand nick at one or both transposon ends generates an open circular product. A double strand break at one transposon end generates the linear plasmid, and a double strand break at both ends excises the transposon and liberates the plasmid backbone. Integration of the excised transposon yields a mixture of inter- and intra-molecular products (29,35).

To interpret the products of the reaction correctly, it is important to recall that the order of nicking events at the transposon ends is kinetically constrained (21). Both 5′-ends of the transposon are normally cleaved before either of the 3′-ends (Figure 4B and Supplementary Figure S2). The rate of strand nicking is therefore 1∼2 > 3∼4. If the transposon ends are cleaved by two active sites in a transposase dimer, there are three models for cleavage, which differ according to which active site cleaves which DNA strand (Figure 4C and Supplementary Figure S3). The simplest case is when one monomer cleaves both strands at the respective transposon ends. In our notation this model would be designated as m/m[1+3/2+4] (Graph 5). The role of each monomer in strand nicking is given before and after the slash. The other models are m/m[1+4/2+3] (Graph 6) and m/m[1+2/3+4] (Graph 7). If the transposon ends are cleaved by the sequential action of four active sites in a transposase tetramer (dimer of dimers), there are also three possible models (Figure 4C and Supplementary Figure S4). The simplest case is when the active sites in one dimer nick both DNA strands at one transposon end. In our notation this would be given as d/d[1+3/2+4] (Graph 2), where the respective roles of the active sites in a dimer are indicated before and after the slash. The other four-active-site models are d/d[1+4/2+3] (Graph 3) and d/d[1+2/3+4] (Graph 4).

If transposition reactions are performed in mixtures containing different ratios of active and catalytically inactive transposases, the six cleavage models predict different distributions of products. Over and above the constraints imposed by the order of strand cleavage, the outcome of the reaction also differs according to whether the active and inactive monomers are recruited into the synaptic complex as homodimers or heterodimers. Heterodimers would arise if active and inactive subunits had been allowed time to re-equilibrate before the start of the reaction. To gain the maximum insight into the mechanism we therefore simulated the distribution of products in each of the two situations (Figure 4C, left and right panels). When there is no pre-equilibration of subunits between active and catalytically-inactive dimers, all of the two-active-site models yield the same distribution of products (Graph 1). Among the four-active-site models, one yields a unique distribution of products (Graph 4), while the other two are indistinguishable (Graphs 2 and 3). A more detailed explanation of the various models and the shapes of the graphs is given in Supplementary Figures S3 and 4.

### Testing the models

In the first transposase mixing experiment, WT and catalytically inactive (D155A) transposases were mixed immediately prior to the reaction. This provided no opportunity for dimers to re-equilibrate (Figure 4D, left panel). The reaction mixtures therefore contained essentially only WT and mutant homodimers. When the substrate and products were plotted, the lines of best fit matched the cleavage model for a single dimer of transposase (Figure 4, compare Graph 1 and Graph 9). This result eliminates all of the dimer-of-dimer models. Indeed, the dimer-of-dimer models predict the accumulation of nicked intermediate in the mixing reactions, which was not observed.

In the second experiment, mixtures of WT and mutant transposase dimers were allowed to re-equilibrate overnight at 37°C before the reaction was started (Figure 4D, right panel). This allowed time for subunit exchange (Figure 1B). The reaction mixtures therefore contained statistical distributions of homodimers and heterodimers, according to the respective ratios of active and inactive monomers. When the substrate and products were plotted (Graph 10), it eliminated the dimer-of-dimer models (Graph 8), in agreement with the first experiment. Indeed, the dimer-of-dimer model predicts the conversion of about 20% substrate to linear product with a peak of linear product at about 20–40% mutant transposase (Graph 8). Although we detected the formation of linear intermediate, its behavior was inconsistent with the dimer-of-dimer model because it peaked at about 50% mutant transposase (lane 19) and did not accumulate above the levels obtained with WT transposase (lane 14). This suggests that the minor fraction of linear products observed originated from other sources including integration events, which have complex behaviors related to the availability of their preferred supercoiled targets (40). Therefore, in agreement with the first experiment, this rules out the dimer-of-dimer models (right panel). However, the experiment could not unambiguously identify the role of each subunit in catalysis. The experimental plot (Graph 10) eliminates the model in Graph 7 but could not distinguish between Graphs 5 and 6, which therefore remain viable.

A final point to note is that the excised transposon fragment did not accumulate in either mixing experiment (Figure 4D). This shows that the subunits involved in transposon excision are subsequently responsible for the integration step. A single active site must therefore perform two cleavage steps followed by one integration step. This experiment does not allow us to determine which pair of strands is cleaved by each active site. That is, it leaves open the question of whether the transposase cleaves both strands at the same transposon end or does it cut one strand at each end? It may seem parsimonious to assume that one monomer would most likely cleave both strands at one transposon end. However, second-strand cleavage is the rate-limiting step in the reaction (35). The long delay between first and second cleavage suggests that there may be a significant structural rearrangement in the synaptic complex.

### A single active site cuts both DNA strands of the same transposon end

**Figure 5.**
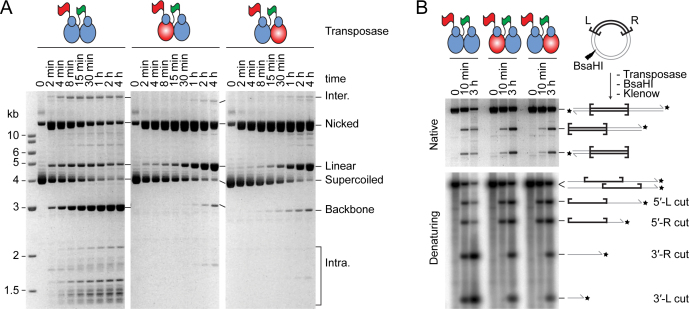
One transposase active site cleaves both DNA strands of the same transposon end. (**A**) Kinetics of transposition reactions were analyzed with WT and mutant single-chain dimer transposases. The mutant single-chain dimers carry an active site mutation (D155A) in one of the subunits (red ovals). Photographs of ethidium bromide stained agarose gels are shown. (**B**) Transposition reactions with WT and mutant single-chain dimers were digested with the restriction endonuclease BsaHI and 3′-radiolabeled with α-[^32^P]-dCTP and the Klenow enzyme. The products were analyzed by native and denaturing agarose gel electrophoresis.

We used the single-chain dimer to further investigate the role of individual subunits within the transpososome. We created derivatives in which one or the other of the subunits was catalytically inactive (D155A). Kinetic analysis revealed that the mutants consumed the supercoiled substrate almost as fast as WT (Figure 5A, central and rightmost panels). This indicates that PEC assembly and first strand cleavage were unaffected in the mutants. However, the mutants accumulated an unusually large amount of the nicked intermediate, which was converted slowly to the linear form. The mutants also produced a trace of backbone product.

Since the mixing experiments have already confirmed the dimer model for cleavage (Figure 4), the accumulation of the linear form indicates that both DNA strands at one end are cleaved by a single monomer (Figure 5A). This demonstrates the veracity of model mm[1+3/2+4] shown in Figure 4B and C. In the mutants, the long delay in converting the nicked intermediate to the linear product reflects the fact that the order of nicks is kinetically constrained, such that completion of both 5′-nicks greatly increases the rate of subsequent 3′-nicks (21).

The timing and levels of linear product accumulation in this experiment can only be explained in the context of the single active site model. Indeed, the linear product was detectable within the first few minutes of the reaction and reached about 50% of total DNA during the course of the reaction. Alternative reactions that could produce this intermediate require dissociation of partially reacted complexes and re-binding of a transposase dimer to a nicked substrate. Both of these processes are slow and inconsistent with the observed kinetics (28,29).

The mutants also produced small amounts of plasmid backbone, which presumably arise from off-pathway reactions (Figure 5A). The most likely explanation is that subunit exchange is not completely suppressed in the single-chain dimer and that two active subunits may yet occasionally come together in a dimer-of-dimers configuration.

Finally, to confirm that the presence of the mutation did not interfere with the ordered cleavage of the 5′- and 3′-ends, the transposition reactions were digested with a restriction enzyme, 3′-end labeled with α-[^32^P]-dCTP and analyzed by denaturing agarose-gel electrophoresis (Figure 5B). As expected, the kinetics showed that the 5′-nicks were chased into 3′-nicks and that this was greatly delayed in the mutants.

## DISCUSSION

**Figure 6.**
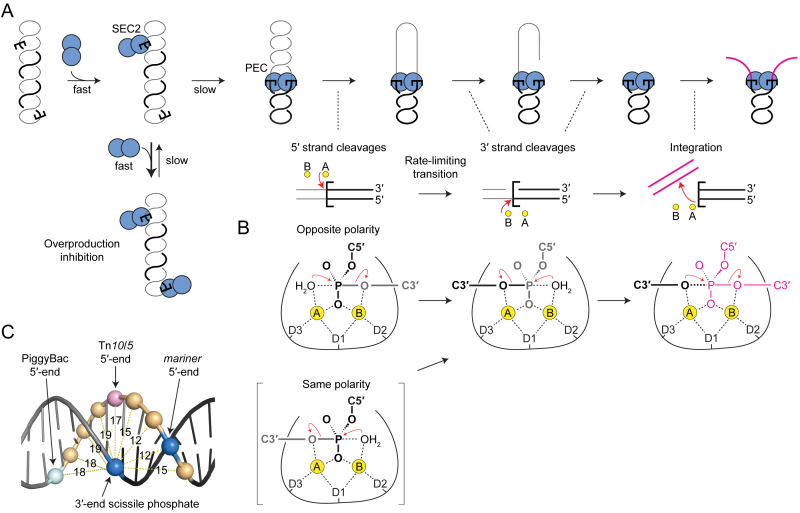
Model of mariner transposition. (**A**) A transposase dimer binds the first transposon ends to form SEC2. If the other end is occupied by another dimer, the two SEC2 compete for recruitment the opposite end, which inhibits the reaction (overproduction inhibition). When the opposite end is free, SEC2 captures the naked end to form the dimeric PEC. Catalysis is initiated by strand nicking at the 5′-ends of the transposon. A structural transition, which is coordinated between the two sides of the transpososome, precedes 3′-strand cleavages. Each active site performs both cleavage events at the same end, followed by transfer of the 3′-end to the target. (**B**) Expected polarity of the DNA strands within the active site and roles of the Mg^2+^ ions during catalysis. See text for details. The active-site residue D155 corresponds to the first aspartate of the catalytic triad (D1). (**C**) A transposon end is illustrated using an idealized section of B DNA. Phosphates are rendered as spheres with distances given in Å. The scissile phosphates in *mariner* are 3 bp apart and are closer (11.9 Å) than any other pair of phosphates on opposite strands. They are almost directly opposite each other across the minor groove. The scissile phosphates in PiggyBac are directly opposite each other across the major groove.

Previously, our biochemical analysis and computer modeling of *mariner* transposition elucidated a mechanism for autoregulation (28). Central to the mechanism is an asymmetric pathway for PEC assembly: a transposase multimer first binds one transposon end and then recruits a second naked transposon end (Figure 6A). Autoregulation is an emergent property of the double occupancy of the transposon ends. The mechanism does not depend on the multimeric state of the transposase, only on the fact that the second end is recruited in an unbound state. Further *in vivo* analysis suggested that the model applies to Sleeping Beauty and PiggyBac in addition to *mariner* (28).

A dimeric model for the *mariner* transpososome was supported by several crystal structures (30–32). However, until now, aspects of the model have remained hypothetical. Namely, the stoichiometry of the pre-cleavage complexes and the dynamics of the reaction, which left scope for significant conformational changes in which subunits exchange positions. Our current results clarify all of these uncertainties. We determined the number of functional subunits and defined the roles of the individual active sites. We have shown that transposition is carried out by a single transposase dimer and that double strand cleavage at the transposon ends are carried out by the same active site. This must be achieved without dissociation and rebinding of the transposase because there is no significant exchange of subunits during the reaction, as revealed by the active and inactive transposase mixing experiments (this work), and because transposon ends remain topologically constrained throughout catalysis, as revealed by the DNA supercoils that are trapped within intramolecular integration products (29). Nevertheless, while protein–DNA contacts are maintained by the transposase DNA-binding and dimerization determinants throughout the reaction, the catalytic domain might still have switched ends between first and second strand cleavage. We have shown that is not the case and that one transposase subunit performs two hydrolysis and one transesterification reactions at each transposon end.

### Two-metal-ion catalysis in a double-strand break nuclease

The active sites of almost all nucleases hydrolyze strands of a single given polarity. Presumably, this arises from interactions surrounding the catalytic center, with non-specific endo- and exo-nucleases taking their cues from the sugar–phosphate backbone. One exception to the polarity-restriction is IS4-family of transposases, which includes Tn*10* and the closely related Tn*5*. These enzymes cleave DNA using a hairpin-mechanism that involves two hydrolysis reactions (12,13). The first hydrolysis is followed by a transesterification reaction, which cleaves the opposite strand and generates the hairpin intermediate. The second hydrolysis resolves the hairpin. However, the polarity of the hairpin intermediate is ambiguous around the apex, and the scissile phosphates in both hydrolysis reactions are attached to the 3′ end of the transposon and are therefore identical.

It is thought that some monomeric restriction enzymes, unrelated to *mariner*, may also be able to cleave strands of opposite polarity. Crystal structures for MspI, BcnI and MvaI suggest that there would be no room for sequence-specific binding of a second subunit (41–44). Since the recognition sites are palindromic or pseudo-palindromic, the simplest mechanism for double strand cleavage would be a cycle of dissociation and rebinding in the opposite orientation. Once the monomeric restriction enzyme has switched strands, cleavage of the palindromic target site is straightforward because the active site is presented with an identical set of protein–DNA interactions. In contrast, transposase have asymmetric recognition and cleavage sites and must therefore accommodate different sets of protein–DNA interactions surrounding the scissile phosphate.

In common with many nucleases and polymerases, the IS4 family and *mariner* use the two-metal-ion mechanism for catalysis (45–47). Yang *et al.* pointed out that the two metal ions are coordinated symmetrically in the Tn*5* cleaved-complex (48). They speculated that this would allow the respective roles of the A and B metal ions to alternate between activating the nucleophile and stabilizing the oxianion leaving group (Figure 6). In the Tn*10* and Tn*5* hairpinning-reactions, this ′ping-pong′ mechanism would minimize the movements of components because the product of one reaction is always the substrate of the next (49).

How does the *mariner* active site deal with the polarity of the DNA strands? The simplest mechanism would be for the active site to rotate ∼40° around the axis of the helix and translocate ∼10 Å along the DNA. The enzyme would have to accommodate different sets of protein–DNA interaction on either side of the scissile phosphates, owing to the different sequence of bases and the opposite polarity of the phosphodiester backbone on each strand. However, this problem arises in any model in which a protein monomer makes a double strand break at an asymmetric recognition site.

The next question regards the role of the metal ions during *mariner* cleavage. The crystal structure of the *Mos1* strand transfer complex suggests that the A metal ion activates the 3′-hydroxyl at the end of the transposon for the integration step (Figure 6B, right element) (32). This is the same as in Tn*5* and the retroviral integrases (50,51). If we now work backward in the reaction pathway: the structure of *Mos1* with an uncleaved transferred strand suggests that the B metal ion activates the nucleophilic water for the cleavage step (31). Once again, this is the same as in Tn*10/5* and suggests that *mariner* conforms to the ping-pong mechanism proposed by Yang (48). Unfortunately there are no uncleaved structures available for *Mos1* or any other cut-and-paste transposon. However, if we continue to be guided by the alternating ping-pong mechanism, it suggests that the A metal ion would activate the nucleophilic water for the 5′-cleavage in *mariner* (Figure 6B, top left). This is the same as in the archetypal, and presumably ancestral, RNase H enzyme (48). In this model the active site of the *mariner* transposase would accommodate the 5′ strand with the opposite polarity to the 3′ strand, which is consistent with our current results and with the simple rotation and translocation of the active site between the scissile phosphates suggested above.

The alternative model is for the active site to engage both strands with the same polarity during hydrolysis. The B metal ion would then activate the nucleophilic water for the first hydrolysis (Figure 6B, bracketed element). The difficulty with this model is that the 5′-strand, or the transposase catalytic domain, has to rotate perpendicular to the axis of the helix to reverse the polarity. For the DNA this would also entail substantial distortion and probably melting. We therefore favor the first model in which the active site moves between the scissile phosphates by a translocation and rotation in the axis of the helix.

It is interesting to note that the scissile phosphates, which are staggered by 3 bp in *mariner*, are positioned directly across the minor groove from each other (Figure 6C). In idealized B-DNA they are 11.9 Å apart, which is the shortest distance between any pair of phosphates on opposite strands. This seems consistent with a mechanism involving sequential hydrolysis reactions because it minimizes the movement of the catalytic components. In contrast, the scissile phosphates in Tn*10/5* are 16.9 Å apart through the middle of the helix. This necessitates separation of the strands for hairpin formation. Unfortunately, there are no structures available for this step, but biochemical analysis shows that bp +2 on the non-transferred strand is flipped following the first nick and before hairpin formation (52–54). Finally, in PiggyBac, another member of the IS4 family, the scissile phosphates are 18.3 Å apart and lie directly across the major groove from each other. It is therefore likely that the hairpin step can be achieved without the aforementioned base flipping.

## Supplementary Material

Supplementary Data
