# Supplementary material for: A single active site in the mariner transposase cleaves DNA strands of opposite polarity
Source: Nucleic Acids Res. 2017 Sep 19;45(20):11467–78. doi: 10.1093/nar/gkx826 (PMC5714172; doi:10.1093/nar/gkx826)
Supplement: Supplementary Data [file gkx826_supp.pdf]

A single active site in the *mariner* transposase cleaves DNA strands of opposite polarity.

Corentin Claeys Bouuaert and Ronald Chalmers

Supplementary Data

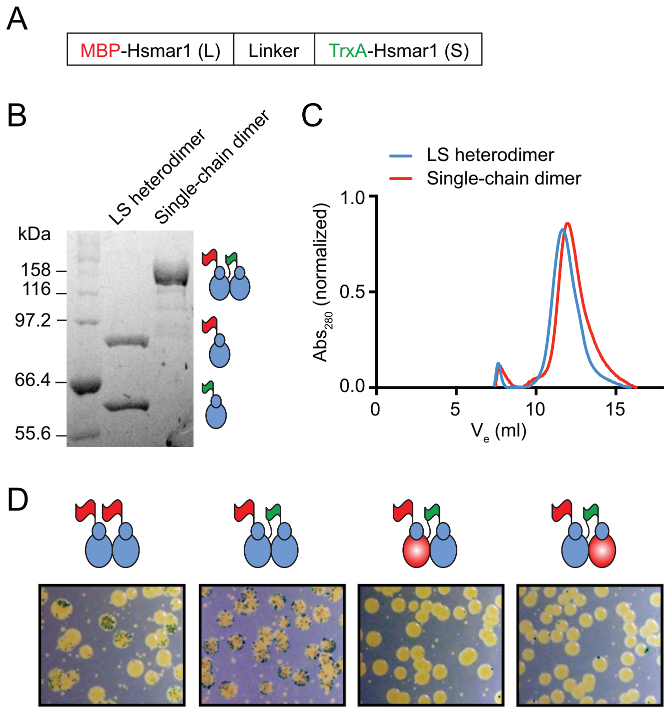

Supplementary Figure S1: A functional single-chain dimer.

(A) The plasmid expressing the LS heterodimer was modified to fuse the C-terminus of the ^{MBP}*Hsmar1* subunit to the N-terminus of the ^{TrxA}*Hsmar1* subunit with a flexible linker (see Materials and Methods). Expression and purification gives a single polypeptide of 1262 amino acids (143 kDa) containing two transposase monomers.

(B) SDS-PAGE analysis of the LS heterodimer and the single-chain dimer.

(C) Gel filtration analysis of the LS heterodimer and single-chain dimer. The similar profiles of the non-covalent and covalent dimers indicate that the single-chain dimer does not form tetramers to detectable levels.

(D) An *E. coli* papillation assay with a non-covalent dimer, the wild type single-chain dimer and mutant single-chain dimers that carry an active site mutation (D155A) in one of the two subunits. The assay relies on the mobilization of a transposon that carries a promoterless LacZ gene (56). Transposition of the reporter construct downstream of an active promoter leads to the appearance of blue papillae that cover the bacterial colonies when grown on X-Gal-containing plates. The experiment shows that the single chain dimer is active in bacteria. When one of the two subunits of the single-chain dimer carried an active site mutation, transposition was abolished. This indicates that the two subunits of the single-chain dimer act together in transposition. A low number of papillae was observed when the second subunit (TrxA-tagged) was mutated. This presumably reflects a low level of dimer-of-dimers in which two WT subunits have come together despite the fact that mass action strongly favors intramolecular dimerization. This is also evident from the larger amount of backbone production with this mutant (Figure 6A, compare centre and right panels). Transposase expression vectors were transformed in the DH5α-based *E. coli* strain RC5096. Transformants were plated on LB-agar medium supplemented with ampicillin (200 µg/ml), lactose (0.1 %) and X-gal (40 µg/ml). Plates were incubated at 37ºC for 5 days and photographed.

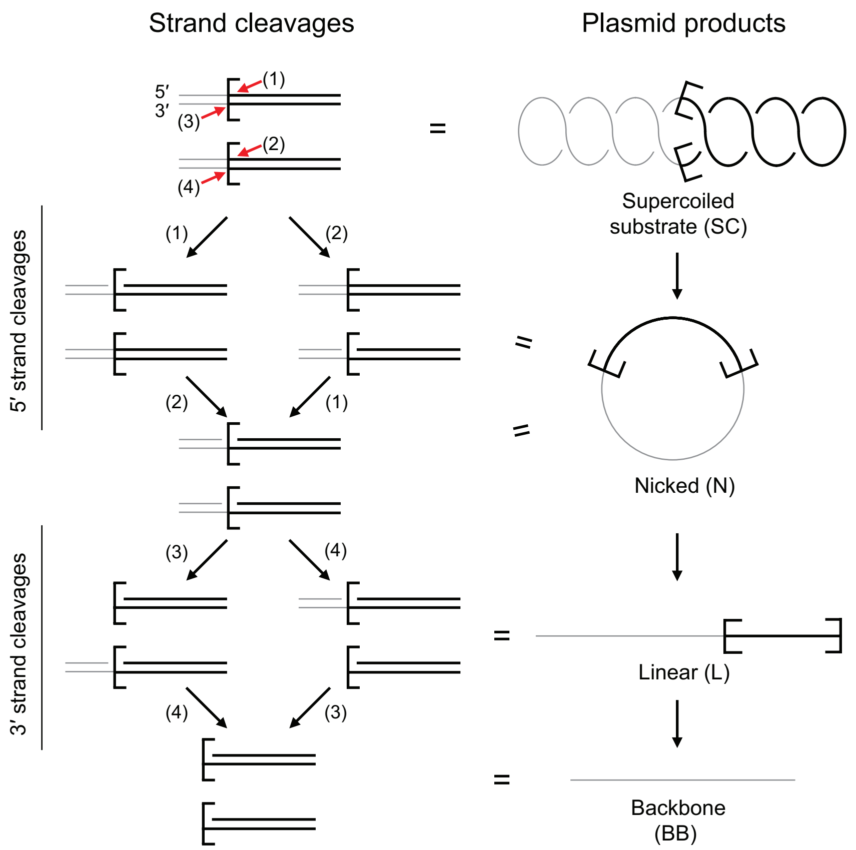

Supplementary Figure S2: Order of cleavage events and products of the plasmid transposition assay.

In the *mariner* transpososome, 5′ strands are cleaved before either of the 3′ strands (21). In a plasmid transposition assay, 5′-end nicks at one or both ends give the nicked intermediate; 3′-end cleavage at one end gives the linear intermediate and 3′-end cleavages at both ends gives the backbone product.

A Dimer model with no pre-equilibration of wild-type (WT) and catalytically-inactive (Mut) subunits

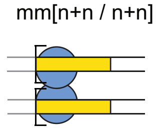

Dimer	WT/WT	Mut/Mut
**Product**	**BB**	**SC**

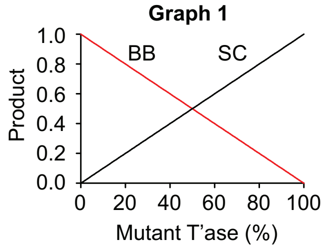

B Dimer models with pre-equilibration of wild-type (WT) and catalytically-inactive (Mut) subunits

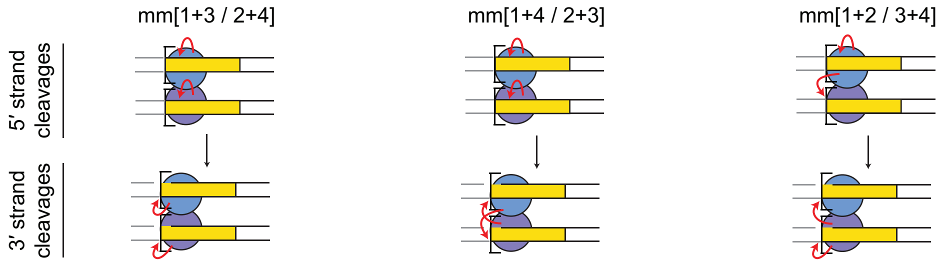

Subunit 1	WT	WT	Mut	Mut
Subunit 2	WT	Mut	WT	Mut
**Product**	**BB**	**N**	**N**	**SC**

WT	WT	Mut	Mut
WT	Mut	WT	Mut
**BB**	**N**	**N**	**SC**

WT	WT	Mut	Mut
WT	Mut	WT	Mut
**BB**	**N**	**SC**	**SC**

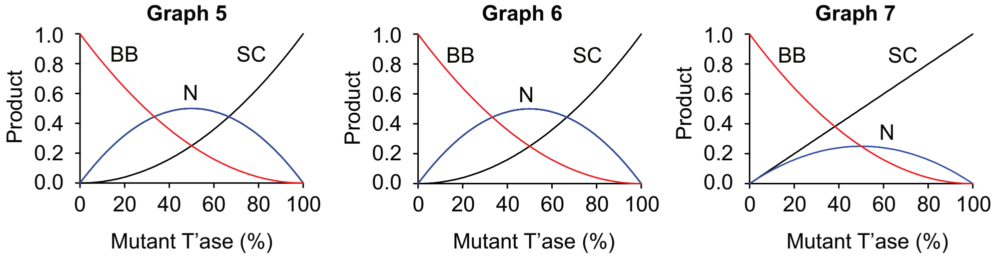

Supplementary Figure S3: Dimer models for transposon cleavage.
For each model there is a defined number of ways in which wild type and catalytically inactive subunits can be arranged on the four DNA strands. The probability of a given combination to occur is given by the equation: $P = [WT]^{n(WT)} \times [M]^{n(M)}$, where [WT] and [M] are the ratios of wild-type and mutant transposases in the mixture, respectively, and n(WT) and n(M) are the number of wild-type and mutant subunits within the combination, respectively. Each combination predicts a specific reaction product from a plasmid assay. The combinations that predict the same outcome are then added up and a graph of the predicted occurrence of each product is plotted as a function of the mutant content in the reaction mixture.
(A) Without subunit pre-equilibration. In this model, the transposon is acted upon by a transposase homodimer. The product outcome is not dependent on the role of individual subunits in the reaction. If the transposase dimer is active, the reaction will give the backbone product (BB). If the transposase dimer is inactive, the supercoiled substrate (SC) remains unreacted. Therefore the predicted outcome of a reaction with increasing ratio of inactive transposase is a gradual decline is backbone and concomitant increase in supercoiled substrate.
(B) With subunit pre-equilibration. In this model, the transposon is acted upon by transposase homodimers and heterodimers. Here, the reaction outcome depends on the role of individual subunits during transposon cleavage, as illustrated. Graphs 5 and 6 are indistinguishable because the coupling of cleavage events in the transpososome is such that 3′ cleavage is greatly reduced when one 5′ end remains uncleaved (21).

A Tetramer models with no pre-equilibration of wild-type (WT) and catalytically-inactive (Mut) subunits

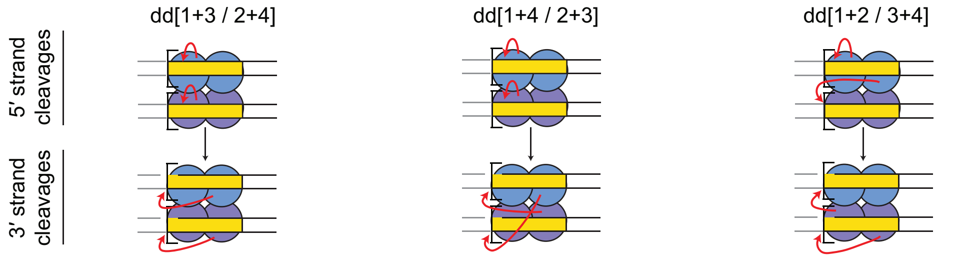

dd[1+3 / 2+4]:

Dimer 1	WT	WT	Mut	Mut
Dimer 2	WT	Mut	WT	Mut
**Product**	**BB**	**N**	**N**	**SC**

dd[1+4 / 2+3]:

WT	WT	Mut	Mut
WT	Mut	WT	Mut
**BB**	**N**	**N**	**SC**

dd[1+2 / 3+4]:

WT	WT	Mut	Mut
WT	Mut	WT	Mut
**BB**	**N**	**SC**	**SC**

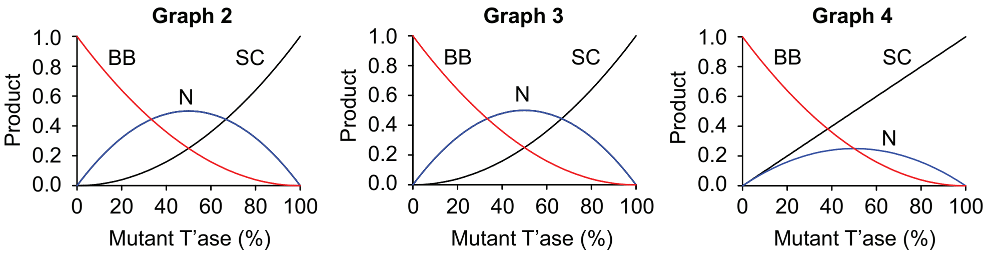

B Tetramer model with pre-equilibration of wild-type (WT) and catalytically-inactive (Mut) subunits

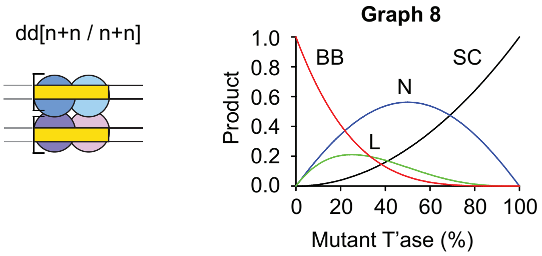

Strand 1	WT	WT	WT	WT	Mut	Mut	WT	Mut	WT	Mut	WT	WT	Mut	Mut	Mut	Mut
Strand 2	WT	WT	WT	Mut	WT	Mut	WT	WT	Mut	WT	Mut	Mut	WT	Mut	Mut	Mut
Strand 3	WT	WT	Mut	WT	WT	WT	Mut	Mut	WT	WT	Mut	Mut	Mut	WT	Mut	Mut
Strand 4	WT	Mut	WT	WT	WT	WT	Mut	WT	Mut	Mut	WT	Mut	Mut	Mut	WT	Mut
**Product**	**BB**	**L**	**L**	**N**	**N**	**SC**	**N**	**N**	**N**	**N**	**N**	**N**	**N**	**SC**	**SC**	**SC**

Supplementary Figure S4: Tetramer models for transposon cleavage.
(A) Without subunit pre-equilibration. In this model, the transposon is acted upon by two transposase homodimers. The products of the reaction depend on which pair of strands each dimer cleaves. Graphs 2 and 3 are indistinguishable because the coupling of cleavage events in the transpososome is such that 3′ cleavage is greatly reduced when one 5′ end remains uncleaved (21).
(B) With subunit pre-equilibration. In this model, the transposon is acted upon by four transposase subunits that behave like independent monomers. Therefore, the reaction outcome does not depend on the role of each subunit. The cleavage products depend on which individual strand is occupied by an active or inactive subunit. There are 16 possible combinations, each of which predicts a specific reaction outcome, which is dictated by the constrained order of strand cleavages in *mariner* (21).

Supplementary Reference

56. Liu, D. and Chalmers, R. (2014) Hyperactive mariner transposons are created by mutations that disrupt allosterism and increase the rate of transposon end synapsis. *Nucleic Acids Res*, **42**, 2637-2645.
